# Scaling friendship

**DOI:** 10.2471/BLT.25.020325

**Published:** 2025-03-01

## Abstract

Can a community-driven mental health initiative be integrated into a government health system without losing its essence? Gary Humphreys reports.

Gogo Dzukwa’s bench is located next to the polyclinic in Hatcliffe, a neighbourhood in Harare, Zimbabwe where permanent homes stand alongside makeshift settlements. Since 2016, she has been talking to people who come and sit on it.

“There is a mix of people in Hatcliffe, but they all know they can come to the bench,” says the 70-year-old Hatcliffe resident. “People may not know me personally, but because I come from the community, they know I will understand many things about their lives, and they know I will listen.”

Dzukwa is one of some 2000 older, female community health workers affectionately known as ‘grandmothers’ who, since 2006, as part of the Friendship Bench initiative, have been trained to provide, among other services, problem-solving therapy, a cognitive-behavioural treatment that helps individuals manage stress, depression and anxiety.

“The grandmothers are mainly addressing depression, which is not only the leading cause of disability worldwide, and a key risk factor for suicide, it is also a leading cause of death among people aged 15–28 years old,” explains Dixon Chibanda, founder and, until recently, chief executive officer of Friendship Bench.

Chibanda piloted the initiative in response to what he considered to be unmet demand for basic mental health care. Like many low- and middle-income countries worldwide, Zimbabwe struggles to meet the mental health needs of its 17.4 million people, providing only two psychiatric hospitals and one psychiatrist per million people.

“The turning point for me was a patient who took her own life because she could not afford the bus fare to get to the hospital,” Chibanda explains. “I knew we weren’t doing enough to get out into the community, and I knew we needed to leverage the power of task-shifting," (a health-care strategy where specific duties, traditionally performed by highly trained professionals, are transferred to less specialized workers).

With a group of colleagues at the University of Zimbabwe, where Chibanda was employed as a clinical psychiatrist and researcher, he started a small task-shifting project in 2006. The core idea was to use older women (the average age of the grandmothers is 76) to deliver talking therapies.

Chibanda had become interested in the role played by such women during a working visit in Benin. “I was fortunate enough to interact with and observe elderly Voodoo priestesses in Ouidah, and I saw the influence the older women had in the community. They were respected – people valued for their opinions and experience.”

As for the bench idea, Chibanda felt that a simple bench in a quiet, communal setting would provide an ideal space for people to share their concerns and stories. “Sitting on a bench is not as intimidating or potentially stigmatizing as going into a clinic, and it is not the same as sitting opposite a person behind a desk,” he says.

“I saw the influence the older women had in the community.”

Dixon Chibanda

Apart from their training in problem-solving therapy, the grandmothers were also trained to refer people for specialist care and schedule consultations. Additionally, they collected data on the smartphones with which they were provided to constitute the evidence base on which the initiative could continue to be developed.

The approach worked, as borne out by multiple studies, including a randomized controlled trial published in the *Journal of the American Medical Association* in 2016, which found that 86% of 286 participants receiving problem-solving therapy from the grandmothers achieved symptom remission after six months, compared to 50% of 287 participants in the control group.

It attracted the attention of several partners, including Grand Challenges Canada (GCC), a non-profit organization funded by the Government of Canada that supports innovative solutions to health, humanitarian and development challenges both at home and abroad in low- and middle-income countries.

“We were very impressed,” says Melani O'Leary, associate director of Global Health Innovation at GCC. “What makes the Friendship Bench extraordinary is not just the evidence-based approach taken by Dixon and his team, but the 'special sauce' – the deep trust, cultural connection and lived experience the grandmothers bring.”

According to Brittney Dudar, portfolio manager for Global Mental Health at GCC, since 2012, GCC has invested some 2.14 million United States dollars (US$) in the initiative, most recently in 2022 when it partnered with the World Health Organization (WHO) Special Initiative for Mental Health, a global effort to improve access to quality mental health services in low- and middle-income countries. “It is one of the most promising mental health innovations GCC has supported to date,” she says.

It is a very successful initiative in a sector where success is hard won, and Chibanda is now handing it over to the Government of Zimbabwe for integration into the public health system. Why?

The simple answer is resources. “The Ministry of Health and Child Care (MoHCC) has the capacity to scale the project and to sustain it, something that my team and I have sometimes struggled to do,” Chibanda says.

According to Alison Schafer, a WHO technical officer with expertise on mental health service delivery in resource-constrained settings, the MoHCC plan is to integrate the Friendship Bench into a broader effort to strengthen community-based mental health and psychosocial support services across Zimbabwe under the National Strategic Plan for Mental Health Services.

Notable in this regard is the FRIENDZ project, that was initiated by the MoHCC in 2023 in collaboration with WHO’s Special Initiative for Mental Health. “One aspect of the Special Initiative is training non-specialist health workers to deliver evidence-based treatments, such as counselling and psychosocial support,” Schafer points out.

Schafer believes that integrating the Friendship Bench into the primary health-care system can help build a comprehensive mental health support system that not only delivers problem-solving therapy but, through strengthened referral pathways and supervision, can ensure (when needed) a smoother transition from primary care to higher levels of mental health services.

As Chibanda points out, referral has been part of the Friendship Bench initiative from the beginning. “Some of the people who come to the bench require specialist care, while many have other health issues, such as human immunodeficiency virus (HIV) and tuberculosis infections,” Chibanda explains, adding that back-referral has also been facilitated, with patients being sent back to grandmothers who help them reintegrate into the community.

Chibanda is hopeful that these functions will be facilitated through integration with the primary health-care system, but he does have some concerns. The first is the degree to which the grandmothers themselves will continue to be involved.

According to Dr Patience Mavunganidze, deputy director of the Mental Health Department at Zimbabwe's MoHCC, the grandmothers will be retained. “The ‘grandmothers’ are village health workers/lay health workers employed by the MoHCC, and they will continue to be,” she says.

“The 'special sauce' [is the grandmothers’] deep trust, cultural connection and lived experience.”

Melani O'Leary

Chibanda hopes that neither the grandmothers nor the benches they sit on will not be lost in the transition. He feels the same way about the language they have introduced. Because, just as the benches brought treatment outside of the clinic, the language used by the grandmothers has brought it out of the – sometimes intimidating – medical lexicon.

Chibanda cites the case of the word “depression” which the grandmothers have replaced with the Shona word “kufungisisa”, which translates to “thinking too much” and is used to describe symptoms of persistent worry, stress and distress.

The grandmothers also gave the project its name. “When we first started, the bench was called the mental health bench,” says Chibanda. “Interestingly, nobody wanted to sit on it. It was the first group of grandmothers who came to me, saying we need to change the name to Friendship Bench. Since then, they have shared their benches, their experience and their empathy with over 624 000 people.”

**Figure:**
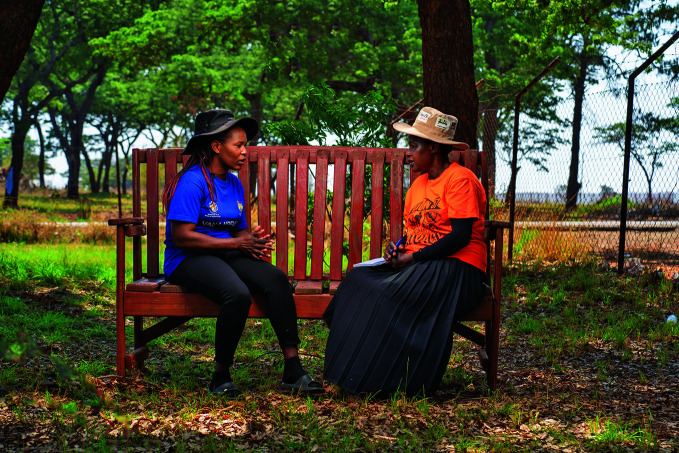
Gogo Dzukwa and client on the Friendship Bench in Hatcliffe

Constantine Juta

**Figure:**
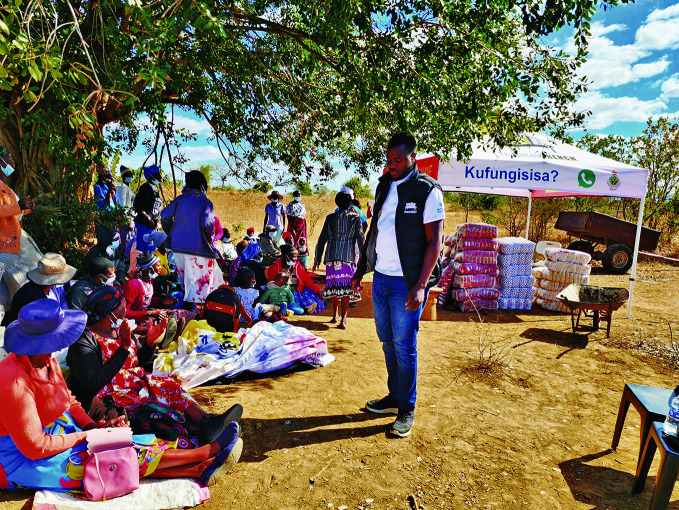
Bringing people together to tackle ‘kufungisisa’

Constantine Juta

